# HPIPS: A High-Precision Indoor Pedestrian Positioning System Fusing WiFi-RTT, MEMS, and Map Information

**DOI:** 10.3390/s20236795

**Published:** 2020-11-27

**Authors:** Lu Huang, Baoguo Yu, Hongsheng Li, Heng Zhang, Shuang Li, Ruihui Zhu, Yaning Li

**Affiliations:** 1 College of Instrumental Science and Engineering, Southeast University, Nanjing 210018, China; hlcetc54@163.com; 2 State Key Laboratory of Satellite Navigation System and Equipment Technology, Shijiazhuang 050081, China; yubg@sina.cn (B.Y.); 13582161539@163.com (H.Z.); lishuangcetc54@163.com (S.L.); zhu_lucking@163.com (R.Z.); 15631149037@163.com (Y.L.); 3 The 54th Research Institute of China Electronics Technology Group Corporation, Shijiazhuang 050081, China

**Keywords:** indoor localization, smartphone, map constraints, sensors, deep neural network

## Abstract

In order to solve the problem of pedestrian positioning in the indoor environment, this paper proposes a high-precision indoor pedestrian positioning system (HPIPS) based on smart phones. First of all, in view of the non-line-of-sight and multipath problems faced by the radio-signal-based indoor positioning technology, a method of using deep convolutional neural networks to learn the nonlinear mapping relationship between indoor spatial position and Wi-Fi RTT (round-trip time) ranging information is proposed. When constructing the training dataset, a fingerprint grayscale image construction method combined with specific AP (Access Point) positions was designed, and the representative physical space features were extracted by multi-layer convolution for pedestrian position prediction. The proposed positioning model has higher positioning accuracy than traditional fingerprint-matching positioning algorithms. Then, aiming at the problem of large fluctuations and poor continuity of fingerprint positioning results, a particle filter algorithm with an adaptive update of state parameters is proposed. The algorithm effectively integrates microelectromechanical systems (MEMS) sensor information in the smart phone and the structured spatial environment information, improves the freedom and positioning accuracy of pedestrian positioning, and achieves sub-meter-level stable absolute pedestrian positioning. Finally, in a test environment of about 800 m^2^, through a large number of experiments, compared with the millimeter-level precision optical dynamic calibration system, 94.2% of the positioning error is better than 1 m, and the average positioning error is 0.41 m. The results show that the system can provide high-precision and high-reliability location services and has great application and promotion value.

## 1. Introduction

Indoor positioning is an extension of navigation positioning technology to the indoor environment. It uses a variety of positioning technologies and sensor information to determine the location of people and objects in indoor space. Outdoors, the direct method of positioning is to use the Global Navigation Satellite System (GNSS), including GPS (Global Positioning System), GLONASS (Global Navigation Satellite System), BDS (BeiDou Navigation Satellite System), etc. However, due to the limited performance of satellite signals in indoor environments, it is impossible to provide high-precision location services in indoor environments. With the increasing demand for services based on indoor locations, such as finding cars in underground garages, rapid medical treatment in hospitals, rapid positioning of materials in factories, etc., more and more research scholars have explored various indoor positioning technologies. According to factors such as application environment, positioning equipment, accuracy requirements, and some users with different needs, the current indoor positioning technology can be divided into professional indoor positioning and lightweight solutions indoor positioning [1]. Common professional indoor positioning technologies include ultra-wideband positioning [2], pseudolite positioning [3], radio frequency identification positioning [4], and ultrasonic positioning [5]. These professional-level positioning technologies are usually required to achieve decimeter or even centimeter-level positioning accuracy, and require high-precision hardware equipment and specific infrastructure. However, when facing the daily applications of the general public, lightweight indoor positioning solutions are a necessary condition. Thanks to the unique popularity and portability of smart phones, smart phones have become a potential mass-level indoor positioning platform. Usually, the low-cost sensors integrated in the mobile phone are used to perceive the user’s movement information and environmental information in real time, and the location of the mobile phone user can be determined. In addition, because smart phones have strong network interconnectivity, smart phone-based indoor positioning has a broader space for development in improving the quality of indoor location services. Generally, the positioning methods based on smart phones mainly include positioning based on wireless sensor networks and the pedestrian dead reckoning (PDR) algorithm. Among them, the most extensively studied include Wi-Fi positioning [6], Bluetooth positioning [7], visual positioning [8], geomagnetic positioning [9], and cellular network positioning [10].

Positioning methods based on wireless signals are favored by researchers because of their advantages, such as wide coverage and mature infrastructure. In particular, positioning methods based on wireless signal location fingerprints have attracted widespread attention. Fingerprint-based positioning technologies are more reliable than connection-based positioning technologies because they do not make any assumptions about communication in the network. However, a pre-configuration stage is required, in which fingerprint collection is performed to model the network to obtain a fingerprint database, that is, a radio map. In the positioning phase, the Radio Signal Strength Intensity (RSSI) collected by any given node is combined with information from the fingerprint database to find the user’s location. The advantage of fingerprint-based technology is that collecting information from the network allows us to consider many characteristics of the environment, such as multipath propagation, wall attenuation, etc. “Location fingerprint” associates a location in the actual environment with a certain “fingerprint”, and a location corresponds to a unique fingerprint. The fingerprint can be single-dimensional or multi-dimensional. For example, when the device to be positioned is receiving or sending information, the fingerprint can be one or more measured values of this information or signal (the most common is signal strength), and any feature that can be used to distinguish positions can be used as fingerprint data, such as carrier phase, pseudo-range, and Doppler in satellite positioning, as well as indoor geomagnetic signals, air-pressure values on different floors, etc. [11]. Commonly used methods include deterministic algorithms, probabilistic algorithms, fingerprint clustering methods, and algorithms based on machine learning. Microsoft first started the research work on Wi-Fi location fingerprint positioning in 2000 and, for the first time, proposed to use the Euclidean distance between the RSS vector and each vector in the fingerprint vector library, to determine the location of the mobile device [12]. The probabilistic location algorithm based on Wi-Fi location fingerprints was first proposed by Reference [13]. The basic idea is that, if you simply use the statistics of an RSS sample (such as the mean value of RSS), it may bring errors, because the actual RSS value should be a distribution. Therefore, the joint probability distribution of multiple APs can be used as a fingerprint, by calculating the probability of all grid points, and then selecting the grid point with the highest probability as the location of the mobile device. There is a problem that is not considered above. Not all grid points can always detect the same set of APs, so the clustering method is proposed in Reference [14]. The basic idea is that the grid points of the same group of APs are considered as a cluster, and the cluster is determined based on the probability of seeing these APs on each grid point, so this method is also called “clustering”. Reference [15] introduced the idea that clustering fingerprints in the signal space can reduce the complexity of fingerprint search. Therefore, he assumed that the same set of APs can be seen in all locations. References [16,17,18] model the relationship between RSS and location information by using deep neural networks and use them to check the match between real-time samples and fingerprint libraries. Because deep neural networks have better learning generalization capabilities, they have achieved better positioning accuracy.

However, the fingerprint-based positioning method is more dependent on the indoor environment, and changes in the environment will affect fingerprint measurement accuracy, resulting in large fluctuations in the positioning results. Therefore, many scholars have proposed the use of filtering methods combined with other sensors to smooth the fingerprint positioning results, and the most used is the combination of inertial navigation technology independent of external infrastructure. Commonly, a wide range of low-cost multi-sensor devices such as smart phones are used to provide information about changes in the state of the user and the environment during the journey, while detecting the relative movement of the user, thereby improving the continuity and accuracy of positioning results. The integration of Wi-Fi fingerprint positioning and pedestrian dead reckoning (PDR) positioning is an effective indoor integrated positioning solution. Generally, the state equation is constructed based on the estimation of the pedestrian position in the PDR equation, and the output position information of the Wi-Fi positioning is used as the observation information to modify and update the navigation solution [19,20,21]. Li et al. [22] considered the pose information of pedestrians in the position tracking algorithm and provided continuous position updates through the PDR algorithm. In addition, the errors in Wi-Fi fingerprint positioning are corrected by sensors such as gyroscopes. Finally, Wi-Fi positioning is passed to the system location tracking module as a metric. Deng et al. [23] used two Extended Kalman Filter (EKF) models to fuse the positioning results of Wi-Fi and PDR. The first measurement model of EKF is based on nuclear density estimation, which can realize accurate Wi-Fi positioning and adaptive measurement noise statistical estimation. The second EKF model is based on the quaternion method, which estimates the heading by fusing the output of the gyroscope and accelerometer. References [23,24] apply UKF and particle filter (PF) to Wi-Fi/PDR system, which achieves a better positioning effect, but the real-time performance is relatively poor. Reference [25] uses adaptive extended Kalman filter to fuse Wi-Fi fingerprint positioning and PDR positioning results, and then uses the fusion results and the wireless signal free space power attenuation model to modify the observation information of the filter to improve the positioning performance of the system.

Although the accuracy and continuity of fingerprint positioning can be improved by filtering and fusion, the characteristics of fingerprint signals are a key factor affecting the positioning results. The commonly used signal strength is extremely susceptible to environmental interference, including indoor temperature and humidity, pedestrian movement and obstruction, and rapid signal fading. Therefore, it is also very important to find a stable and robust fingerprint signal. At present, Wi-Fi round-trip time (RTT) location is a kind of location method based on round-trip time ranging, and Wi-Fi RSSI location is more based on signal fingerprint matching. The measurement technology based on Wi-Fi RTT can theoretically achieve an accuracy of 0.1 ns for time measurement, that is, a range accuracy of about 3 cm. Although limited by multipath factors, accurate ranging in complex environments such as non-line-of-sight cannot be achieved, the ranging information obtained through the fine time measurement (FTM) technology is relatively stable and has obvious spatial position discrimination, which meets the characteristics of fingerprint information [26]. In response to the above problems, we combined the characteristics of fingerprint positioning and the advantages of FTM ranging, and used the deep learning network to learn the mapping relationship between spatial line-of-sight/non-line-of-sight geographic locations and ranging information to build a positioning model. Then, in response to the problem of real-time position jump caused by the fluctuation of the ranging information in absolute positioning, a nonlinear particle filter model is introduced to fuse sensors related to the PDR algorithm and a priori map information to constrain the user’s position. Finally, a high-precision, high-stability continuous absolute positioning system (HPIPS), was realized on the smartphone. The specific contributions of this article are as follows:

1. A deep convolutional neural network model is proposed to learn the mapping relationship between indoor spatial location and Wi-Fi RTT ranging information. In the dataset construction stage, the indoor area is divided into equally spaced grids, and each grid corner is used as a sampling point. At the same time, the collected data are visualized as a gray image with ranging information and actual AP location information. Then, the data features are extracted through the convolution operation of each layer for position prediction. Finally, the experimental results show that the proposed positioning model has higher positioning accuracy than common positioning algorithms based on fingerprint matching.
2. Aiming at the problems of poor stability and low accuracy of positioning results, a method of fusing the positioning results of Wi-Fi models, sensors related to the PDR algorithm information, and structured indoor map information, using an adaptive particle filter algorithm, is proposed. The microelectromechanical systems (MEMS) sensor in the smartphone can estimate the motion state of the pedestrian, and adaptively update the particle filter state transition equation, thereby improving the degree of freedom and stability of pedestrian positioning. At the same time, combined with indoor priori map information to restrict pedestrian trajectories, and further improve the positioning accuracy and stability of the positioning system.
3. In order to verify the positioning performance of the HPIPS system, a large number of experiments and performance analysis work were carried out in an experimental environment of about 800 square meters, and the positioning accuracy was compared with millimeter-level optical calibration systems and commonly used positioning algorithms. The experimental results prove that the constructed indoor positioning system can provide users with stable, reliable, continuous, and high-precision absolute position information, which has certain popularization and application value.

The remainder of this paper is organized as follows: In the second section, some previous work related to the research content of this paper is summarized, and the basic principles of FTM ranging and filtering fusion are also introduced. In the third section, the system framework and workflow proposed in this paper are described. The fourth section introduces the indoor positioning technology based on convolutional neural network and the fusion details based on particle filter. In Section 5, we verified the positioning performance of the proposed system through a large number of experiments. In the last section, we summarized all the work of the article, discussed the advantages and limitations of the system, and looked forward to some work to be done in the future.

## 2. Preliminaries and Overview of System

### 2.1. Wi-Fi FTM

**Figure 1:**
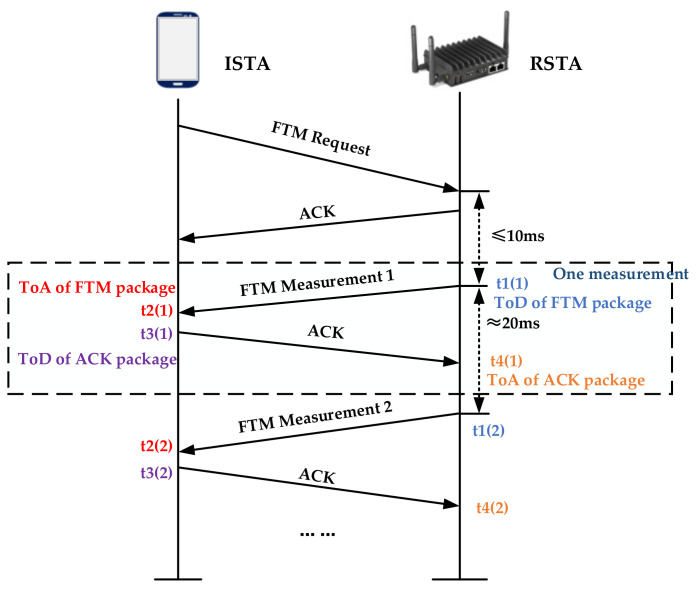
Schematic diagram of the ranging process of Wi-Fi fine time measurement (FTM).

**Figure 2:**
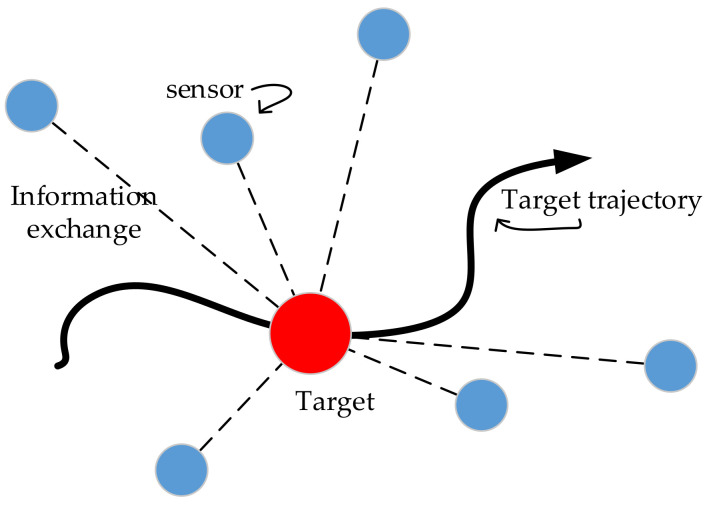
Filter-based target tracking and positioning process.

IEEE 802.11mc-2016 standardized FTM protocol [27], which enables a pair of Wi-Fi base stations to estimate the distance between them. Figure 1 shows the principle of FTM ranging. Ibrahim M and his partners analyzed the key factors affecting Wi-Fi ranging performance based on the open platform, and revised the standard error correction technology for Wi-Fi FTM-based positioning systems [28]. FTM is a point-to-point (P2P) single-user protocol, which includes the exchange of multiple message frames between the initiator station (ISTA) and the receiving station (RSTA). RTT is calculated based on the time stamp captured when the FTM frame leaves and arrives. The protocol itself is similar to the previous 802.11v timing measurement protocol. One of the most significant improvements is the increase in time stamp resolution from 10 nanoseconds to 100 picoseconds. During the FTM process, ISTA starts an FTM session with RSTA. One ISTA can initiate multiple sessions at the same time (for example, one ISTA measures the distance to multiple APs, or one AP responds to multiple ISTAs). FTM conversation includes three stages in total: negotiation, metric exchange, and termination, the details of which are shown in Figure 2. First, ISTA sends an FTM request to RSTA and waits for its ACK message. Then, RSTA receives the FTM request and sends an ACK message back to ISTA. Then, multiple RTM feedbacks are sent from RSTA to ISTA, and RSTA records the ToD (departure time) t1 (1) of the FTM packet. Once ISTA receives the ToA (time of arrival) t2(1) of the FTM packet, it will also be measured. The process of ACK packet exchange is similar. After recording ToD t3 (1) and ToA t4 (1), an FTM exchange has been completed. If the ACK exchange of FTM is performed n times, (1) can be used to estimate the average round-trip time [29]:

(1) $$RTT=\frac{1}{n}\left(\sum_{k=1}^{n}t4\left(k\right)\;-\sum_{k=1}^{n}t1\left(k\right)\right)\;-\frac{1}{n}\left(\sum_{k=1}^{n}t3\left(k\right)\;-\;\sum_{k=1}^{n}t2\left(k\right)\right)$$

After obtaining the average round-trip time, the distance between ISTA and RSTA can be obtained by multiplying the time by the speed of light. The theoretical ranging model can be defined as follows:

(2) $$Range=RTT\times \frac{C}{2n}$$

Among them, $C=3\times 10^{8}m/s$ is the propagation speed of the electromagnetic signal. In order to provide a more accurate RTT estimation, the FTM interaction can usually be repeated multiple times in the form of a pulse train. The smartphone in Figure 1 needs to meet the operating system above Android Pie and support the FTM (IEEE 802.11mc) protocol, such as the Google Pixel series of mobile phones.

### 2.2. Filtering Technology in Target Tracking

To address the problem of state estimation of the target to be located, one of the common methods is Bayesian filtering, which provides a general framework for estimating the state of the system based on its observations [30]. Usually a state and the state-related confidence coefficient (such as the form of a covariance matrix) is used to estimate the system state. The first step in performing target tracking and positioning is usually to build a model that describes the target’s movement. Once the previous state is known, the motion model (also called the state space model) can be used to predict the actual state. For example, if you know the location, speed and direction of a driving vehicle, you can use the state-space model to predict its current location. However, the longer the forecast range, the greater the uncertainty of the estimate. Therefore, this uncertainty also needs to be included in the state space model. The second step is to update the predicted value using other received information. This information can be any relevant observations collected from the positioning network. In Figure 2, the process of exchanging the target with various sensors in the network while moving is described. A relationship must be found between the required location and the collected metrics to modify the model, that is, we need to define a metric model (observation model).

Of course, various uncertainties must be considered in this model. Generally, depending on the type of measurement, an appropriate filtering method can be selected to balance the information from the state space model and the observation model. For example, if the observation model is linear, the Kalman filter can be used [31]. The filter first uses the previous estimated position and the state space model to predict the unknown position. Then, the observation model is used to correct the predicted position. The main limitation of the Kalman filter is that it is only reliable for almost linear systems. In order to solve the problem of nonlinear estimation, methods based on extended Kalman filter (EKF) [32] and unscented Kalman filter (UKF) [33] have been proposed by researchers. However, the main disadvantage of these methods is that they perform linearization and approximation, resulting in suboptimal performance and sometimes divergence of the filter [34]. In particular, since EKF is essentially a linear approximation of the observation equation, it can hardly achieve a good approximation of a highly nonlinear observation model.

Aiming at the problem of nonlinear and non-Gaussian target tracking and positioning, the famous particle filter (PF) was proposed by Branko Ristic, in 2004 [35]. Particle filtering is also called Monte Carlo filter and relies on the minimum mean square error estimation of the target state. It attempts to represent the posterior distribution of the hidden state through a large number of appropriately weighted random samples that change over time. When the number of samples reaches infinity, in a certain statistical sense, the weighted average of these samples will converge to the true estimate of the currently unknown state [36]. For this type of filter, the observation model can be non-linear, and the initial state and noise distribution can take any desired form. Compared with the Kalman-based method, this algorithm requires more calculations due to particle generation and resampling, but it has greater potential when the noise is not Gaussian and the model is nonlinear [37]. Moreover, due to the improvement of computer hardware level, the iterative calculation of a large number of particles has been efficiently realized on smart phones, making real-time target tracking and positioning possible.

## 3. System Overview

**Figure 3:**
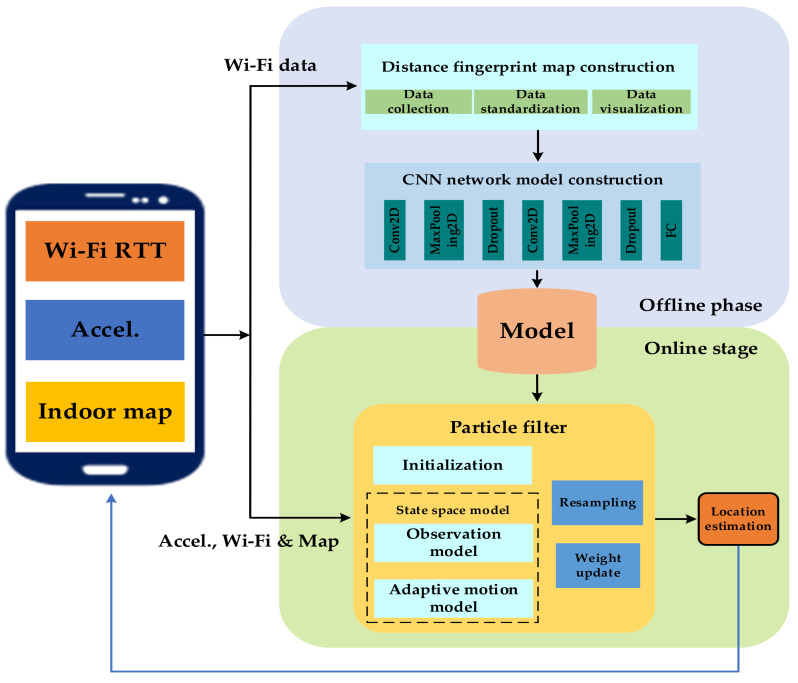
Structure diagram of high-precision indoor pedestrian positioning system (HPIPS).

The HPIPS positioning system designed in this paper is composed of Wi-Fi RTT devices and smart phones that support the IEEE 802.11mc protocol, at present, Google pixel and Samsung mobile phones already support this agreement. In the fingerprint positioning technology based on smart phones, the traditional way of searching and matching using fingerprint database cannot meet the real-time requirements of positioning due to searching too much fingerprint data, and it increases the burden of system energy consumption. Therefore, in order to improve positioning efficiency, a positioning framework, as shown in Figure 3, is proposed. The fingerprint dataset is trained by machine learning methods to extract representative important features and output these feature parameters in the form of a model. In this way, the built-in deep learning positioning model can quickly respond when users need to locate, and achieve real-time positioning effects. In the offline phase, the fingerprint data of the signal are first collected in the area to be located, and a fingerprint database is constructed. Then the preprocessed dataset is trained through the designed deep learning network model, and the positioning model is obtained and loaded into the positioning software to perform the real-time execution of the model. In the online phase, using the HPIPS application software installed on the smartphone, users can achieve rapid positioning in the indoor building environment without any operation.

## 4. Proposed Method and Implementation Details

### 4.1. RTT Fingerprint Location Technology Based on Convolutional Neural Network

#### 4.1.1. Overview of Basic Ideas

After consulting relevant information and experimental tests, it is found that the ranging error of RTT does not follow Gaussian distribution, but a discrete value related to location. For direct positioning based on RTT ranging, it is difficult to ensure that each position can achieve a good positioning estimation, especially under the influence of non-line-of-sight and multipath. Therefore, we use deep convolutional neural networks to learn as much as possible the mapping relationship between the location and the RTT ranging fingerprint information database, and find stable and reliable feature information to achieve pedestrian location estimation in an indoor environment. Among them, the convolutional neural network (CNN) model is a deep network, which can be used to understand the data from the spatial structure of the data. A typical CNN network generally consists of three parts: convolutional layer, pooling layer, and fully connected layer. Through the free combination of the above three parts and the adjustment of parameters and structure, different CNN models can be constructed to solve different problems. The fingerprint image constructed by Wi-Fi RTT ranging information is used as the input of the convolutional layer of the CNN network. Among them, it is necessary to convert the two-dimensional data in the original fingerprint database into three-dimensional data and realize the image processing of the data as the input of the CNN network model, and the output as the position coordinates. The convolutional layer understands the characteristics of the fingerprint image by identifying the spatial relationship between different pixels in the image. When preprocessing the input image, our method is different from the traditional one-dimensional data directly converted into a two-dimensional image, but when each image is constructed, the specific location information of the AP is combined to fill in the fingerprint data. In this way, a two-dimensional grayscale image that conforms to the real physical meaning is constructed, and when the convolutional neural network is trained, more representative features are extracted to facilitate the learning of the model.

#### 4.1.2. Description of Algorithm Details

##### Building Training Datasets

**Figure 4:**
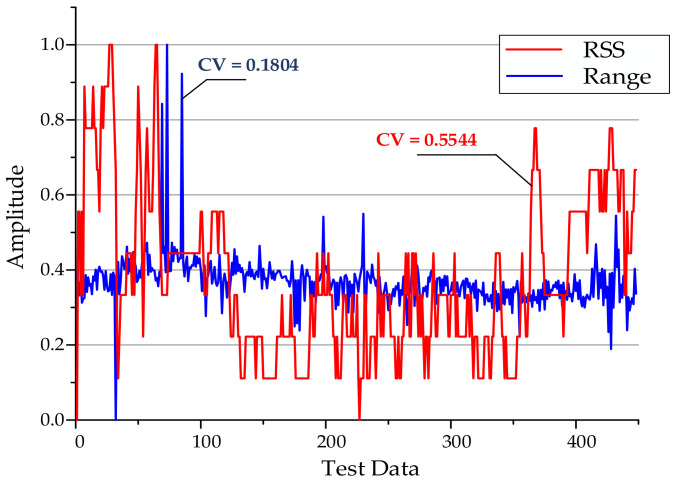
Compare the fluctuation of ranging and Radio Signal Strength Intensity (RSSI) by coefficient of variation.

Generally, researchers choose the signal strength of AP to build a fingerprint database. However, in indoor environments, the signal strength is severely affected by the environment, and short-distance signals are quickly fading, which often results in a large range of signal fluctuations at the same location. Figure 4 shows the comparison data of signal strength value and ranging over a period of time. When the user is stationary in the same location, it reflects the changes of the received RSS and RTT ranging information over time. In order to improve the convergence speed and classification accuracy of deep learning networks, normalized preprocessing is usually performed on the data input to the network. At the same time, the coefficient of variation (CV) is used to measure the degree of variation of observations with different dimensions. The calculation method of CV is ratio of standard deviation to average. In this article, we have compared and analyzed the dispersion degree of the preprocessed ranging information and RSSI for the same AP, the same receiving device at the same location, over a period of time. It can be seen from the figure that the dispersion degree of using RSSI to construct environmental fingerprints is much greater than that of ranging fingerprints, where CV-RSS = 0.5544 and CV-RTT = 0.1804. If RSSI is used to construct a fingerprint database, it will increase the difficulty of learning the network model, resulting in poor positioning effect. Therefore, from the perspective of fluctuation degree and model training, FTM’s ranging information is used for fingerprints, which can improve the convergence speed of the network and is more suitable for positioning.

In the offline dataset construction stage, the existing AP equipment in the structured indoor environment is usually used as the signal source, but it is uncertain whether the location of the public AP is often moved or the signal is often blocked. Therefore, in response to the above problems, we choose to deploy some APs that support Wi-Fi RTT in the environment to be located, and measure the location of the APs through a total station. Taking into account factors such as deployment cost and positioning accuracy, the number of APs usually deployed is limited. To achieve a better positioning effect, the traditional fingerprint database construction method must be improved. Therefore, in view of the characteristic that the CNN network can learn the structural characteristics between data, a fingerprint database construction method based on the known real AP location is proposed. The constructed fingerprint map does not only reflect the RTT ranging information of each location, but also reflect the location of the AP in the real environment. Specific steps are as follows.

**Figure 5:**
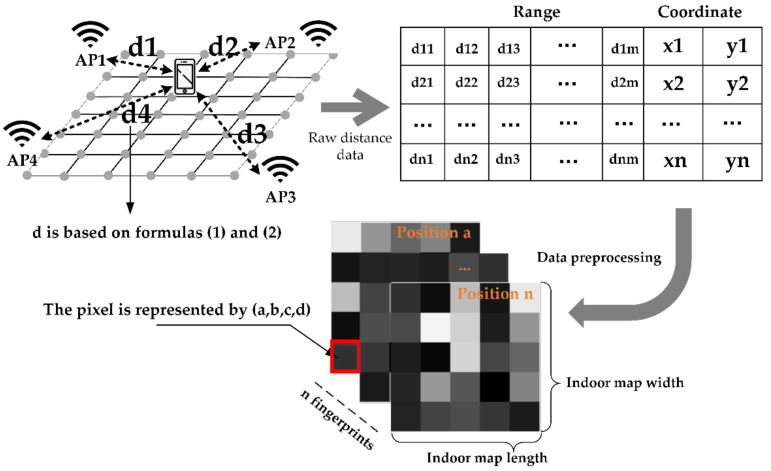
Dataset construction method.

First, deploy APs in the indoor environment to be positioned, with the principle of maximum visibility. Then, the area to be positioned is divided into equally spaced positioning grids, and the grid size is determined according to the positioning accuracy requirements. The experimenters collected the distance measurement information in different directions from the north, south, east, and west at the corners of each grid, and, at the same time, formed fingerprint data with the coordinates of the corresponding corners. Finally, define each piece of fingerprint data as an image with a fixed length and width (the length and width here can be determined according to the size of the specific scene to be positioned). Fill in the corresponding distance information according to the location coordinates of the AP, where the depth of gray represents the strength of the distance fingerprint. The concrete realization is shown as in Figure 5. In the figure, m is the number of deployed APs, and n is the number of sampling points. Each square in the grayscale image represents the distance information between the current location and each AP. Calculate the distance between different sampling points and each AP based on Formulas (1) and (2), where d21 represents the distance from position 2 to AP1. For example, as shown in Figure 5. Among the pixels numbered (a, b, c, and d), a represents the location label, b represents the abscissa of the AP, c represents the ordinate of the AP, and d represents the standardized value of the distance between the location a and the AP.

##### Build Indoor Positioning Model

Convolutional neural network is a feed-forward neural network inspired by biological natural visual cognitive mechanism. In general, the input image matrix, convolution kernel, and feature map matrix are all square matrices. We set the input matrix size to ω, the convolution kernel size to K, the stride size to s, and the number of zero-padded layers to P. Then the calculation Formula (3) for the size of the feature map $\omega^{\prime }$ generated after convolution is calculated as follows:

(3) $$\omega^{\prime }=\frac{\left(\omega +2p-K\right)}{s}+1$$

After the feature map is obtained, pooling (also called sub-sampling) is usually required to reduce the amount of data. Like convolution, pooling also has a sliding kernel, which can be called a sliding window. Usually, max pooling or mean pooling can be used to compress the data in the sliding area to reduce the complexity of the model. Connect the output of the pooling layer to the fully connected layer for final classification or regression. Suppose we train a multi-channel image V, define this training process as $c\;\left(K,V,s\right)$, and minimize the loss function $J\;\left(V,K\right)$. In the forward propagation process, we need to obtain the output intermediate quantity Z through c, and then Z is passed to the rest of the network and used to calculate the loss function J. In the process of backpropagation, we will get a tensor G that satisfies Formula (4):

(4) $$G_{i,j,k}\;=\;\frac{\partial }{\partial K_{i,j,k,l}}J\;\left(V,K\right)\;=\;\sum_{m,n}G_{i,m,n}V_{j,\left(m-1\right)\times s+k,\left(n-1\right)\times s+l}$$

If this layer is not the last layer of the network, the gradient of V needs to be calculated by Formula (5), so that the error is further propagated back.

(5) $$\begin{array}{ll} h{\left(K,G,s\right)}_{i,j,k} & =\frac{\partial }{\partial V_{i,j,k}}J\;\left(V,K\right)\\ & =\sum_{\begin{array}{l} \;\;l,m\\ \;\;s.t.\\ \left(l-1\right)\times s+m=j \end{array}}\sum_{\begin{array}{l} \;\;n,p\\ \;\;s.t.\\ \left(n-1\right)\times s+p=k \end{array}}\sum_{q}K_{q,i,m,p}G_{q,l,n} \end{array}$$

**Figure 6:**
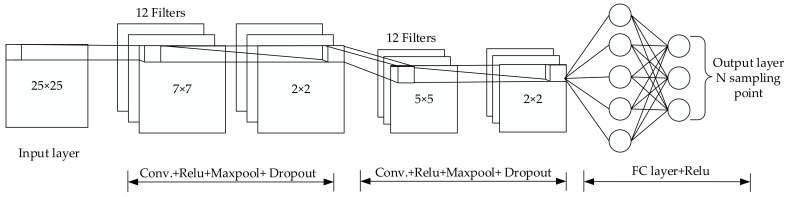
Convolutional neural network (CNN)-based indoor positioning model.

In the above formula, i represents the i-th output channel, j and k represent the output row and column, the input channel is l, and the input row offset term and column offset term are M and N, respectively. Generally speaking, in the conversion process from input to output, a non-linear operation is realized by adding a bias term to each channel, and this bias term can be shared in each convolutional layer [38]. In this article, the deep learning library keras [39] serves as a network model building tool. Generally, the construction steps of keras include the following: model selection, network layer construction, compilation, training, and prediction. In the construction of the network layer, a convolutional neural network including an input layer, a convolutional layer, a pooling layer and a fully connected layer is designed. In this article, according to the size of the actual test scene and the actual location of the AP deployment, multiple 25*25 images are constructed as input data. The specific construction method is as follows: First, 12 7*7 convolution kernels and 2*2 maximum pooling (strides = 1) are used to convolve the image, and the part smaller than the size of the convolution kernel is discarded. Then, using the 5*5 convolution kernel, the maximum pooling of 2*2 (step size = 1) is performed again for the convolution operation. Finally, the structure of the pooling layer is flattened and then connected to the fully connected layer for location prediction. Among them, ReLU is used as an activation function. Its advantage is a piecewise linear function, which belongs to a unilateral inhibition function, and can make neurons have sparse activation. At the same time, in order to prevent the training from overfitting and the trained model from being too concise, the Dropout layer is usually added after the fully connected layer, and the value is 0.5, and the learning rate is set to 0.001. The specific model structure is shown in Figure 6. The back propagation algorithm is used to train the entire network, and when the loss function between adjacent iterations drops below the threshold or the number of iterations is met, the network parameters are saved and the training is stopped. Generally, a cross-validation method is adopted, and training is repeated until the model converges. Finally, the trained model is stored in the memory of the smartphone for real-time fingerprint positioning.

**Figure 7:**
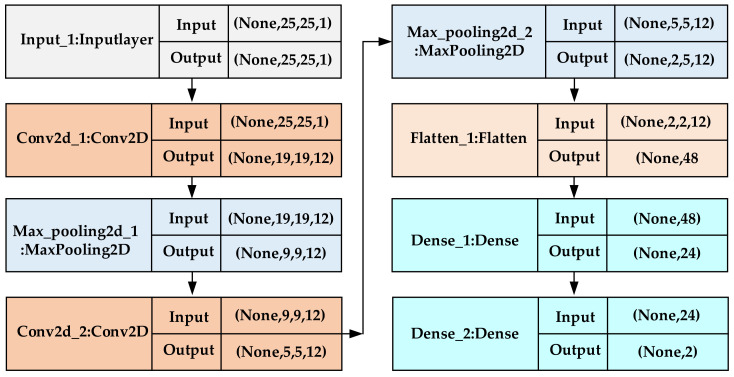
Visualization of positioning model structure.

The keras model function is used to visualize the structure of the model, including the number of layers and the parameters of each layer, as shown in Figure 7.

### 4.2. Multi-Information Fusion Positioning Algorithm Based on Particle Filter

Considering that in the real environment, there are often many interference factors in radio signal propagation, such as signal reflection, refraction, or diffraction, which leads to the unsatisfactory effect of using the wireless signal fingerprint library for positioning. Therefore, a particle filter method that uses excellent performance in nonlinear and non-Gaussian problems is introduced to fuse the multi-sensor information in the smart phone and the map information in the structured space environment to smooth the positioning results of the CNN network model. Thereby further improving the stability and continuity of the positioning results. The implementation details of particle filter fusion of multiple information sources will be introduced in this section. In the prediction process of the particle filter algorithm, some state transition rules are set for particles based on experience, such as moving each particle at a uniform speed, and estimating the possible position of the particle at the next moment according to the change of the real building environment. It is also possible to define more detailed randomness to simulate various motion states. Therefore, particle filtering is more suitable for indoor positioning applications where the state distribution is unknown and cannot be formulated. Generally, dynamic systems can be described by state models and observation models, as shown in Formulas (6) and (7):

(6) $$X_{k}=f_{k}\;\left(X_{k-1},W_{k}\right)\;W_{k}\sim N\;\left(0,Q_{k}\right)$$

(7) $$Z_{k}=h_{k}\;\left(X_{k},V_{k}\right)\;V_{k}\sim N\;\left(0,R_{k}\right)$$

where $X_{k}\in {\mathbb{R}}^{n_{x}}$ and $Z_{k}\in {\mathbb{R}}^{n_{z}}$ are the state value and observation value of the system at time k, respectively. $W_{k}\in {\mathbb{R}}^{n_{w}}$ and $V_{k}\in {\mathbb{R}}^{n_{v}}$ are process noise and measurement noise, which are usually considered Gaussian noise with zero mean and covariance matrices $Q_{k}$ and $R_{k}$.

$f_{k}:{\mathbb{R}}^{n_{x}}\times {\mathbb{R}}^{n_{w}}\to {\mathbb{R}}^{n_{x}}$ is a nonlinear function that reflects the relationship between the current state and the previous state, and $h_{x}:{\mathbb{R}}^{n_{x}}\times {\mathbb{R}}^{n_{v}}\to {\mathbb{R}}^{n_{z}}$ represents the relationship between the observed value and the state [40]. The basic process is as follows.

1. Initialization: The initialization phase includes two parts, namely particle state space initialization and deep learning positioning model initialization. $H=\left\{x^{i}\left|i=1,2,\cdots ,n\right.\right\}$ is the particle set, where n is the number of particles, which is determined according to the positioning accuracy requirements and the real-time requirements of the system. In this article, the number of particles is determined by the software-side algorithm running time and positioning accuracy. For example, after selecting 2000 particles, it takes about 100 ms to test the algorithm operation on the Android software side, which meets the real-time positioning requirements. The particle state space contains position coordinates and movement steps. The initialization of the deep learning positioning model generally constructs the same network model as the training before the positioning is executed, and loads the model parameters and interfaces.

2. Position prediction: The PDR algorithm is used as the state transition equation of particles to realize the prediction of particle position, as shown in Formula (8).

(8) $$\left[\begin{array}{l} x_{k}\\ y_{k} \end{array}\right]=\left[\begin{array}{l} x_{k-1}\\ y_{k-1} \end{array}\right]+\left[\begin{array}{l} L_{k}•\sin\theta \\ L_{k}•\cos\theta \end{array}\right]$$

Among them, the position coordinate at time k−1 is $\left(x_{k-1},y_{k-1}\right)$, the position coordinate at time k is $\left(x_{k},y_{k}\right)$, and the distance traveled at time before and after is $l_{k}$. At this time, the moving distance of each particle is calculated by the self-contained sensor in the smart phone and the step-length estimation model to achieve adaptive motion. θ is the moving direction of the particles. Here, since the accuracy of the direction sensor is relatively low, we do not restrict the direction to ensure the diversity of particle states. The specific description is as follows.

(1) When pedestrians are walking, acceleration information is composed of three components: horizontal, vertical, and lateral. When pedestrians place their smartphones horizontally in front of them, they correspond to the *y*, *z*, and *x* axes of the mobile phone coordinate system. The common method to detect walking is the peak detection algorithm. Because the acceleration information in the vertical direction will change periodically, the difference in acceleration is used to detect the peak and valley values that meet the threshold limit within one step, so as to obtain the number of pedestrian steps.

(2) Step length estimation methods are generally divided into two types: One is to set a constant value as the pedestrian step length according to the pedestrian’s height and weight, and the other is to establish an adaptive step length calculation formula based on walking characteristics [41]. Although the latter is more complicated, its accuracy will be improved to a certain extent, as compared to the former. This research adopts the latter algorithm and calculates the step length by Formula (9).

(9) $$L=K\left(\left(a_{\max}-a_{\min}\right)\times 3.5+\sqrt[{4}]{a_{\max}-a_{\min}}\right)$$

Among them, K is the step length estimation parameter, which is determined by the statistical analysis of the measured data; $a_{\max}$ and $a_{\min}$ correspond to the maximum and minimum values of the acceleration during a single step.

(3) Direction estimation generally uses an electronic compass for testing. In order to increase the diversity of particles, we choose to move the particle set in random directions to improve the reliability of posterior probability estimation.

Through the above three steps, we will update the step size attributes of each particle in real time to further improve the degree of freedom of the system positioning. In the next section, we also did a comparative test to verify the effectiveness of the adaptive step size motion model.

**Figure 8:**
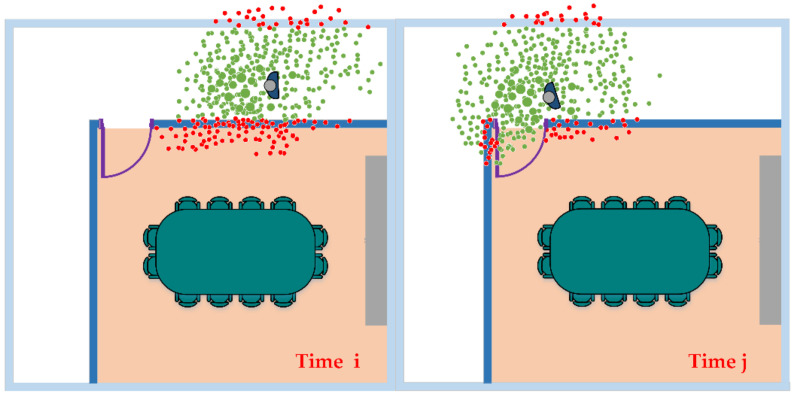
Schematic diagram of map constraints.

3. Weight update: Compare the predicted value with the probability distribution function obtained from the actual measurement process to update the weight. Similarly, in this article, the CNN network model is used as an observation function to calculate the observation position through real-time fingerprint data. Then, the Euclidean distance between each new particle and the Wi-Fi RTT fingerprint positioning result is used to update the weight. The larger the weight, the closer to the true position. Finally, calculate the weight of each particle by using Formula (10):

(10) $$\omega =\frac{1}{\sqrt{2\pi }\sigma_{\omega }}\exp\left\{-\frac{{\left|s-g\left(\delta \right)\right|}^{2}}{2\sigma_{\omega }{}^{2}}\right\}$$

where s is the particle state at the current moment, $g\left(\delta \right)$ is the particle observation state obtained through the deep learning positioning model, and $\sigma_{\omega }$ is the measurement deviation. After all particles have weights, particles with low weights need to be filtered out. These particles are considered to be far from the user’s real state. The purpose of resampling is to concentrate the particles in the vicinity of the high-weight particles so that the particle swarm can converge. However, in practical applications, some particles will “through the wall” phenomenon during the state update process. Therefore, the real geographical environment information should be considered in the stage of updating the weight. For indoor positioning, building plans are very useful information, which can be used to improve location accuracy and reduce the uncertainty of walking trajectories, and particle filters are often used as a “map filtering” technology to consider building plan information. In this article, combining the corridor boundary and doors and windows and other architectural structures, restrict the position distribution of the particle swarm, and reset the weight of the particles through the wall to zero. As shown in Figure 8, the blue point is the position where the user may move in the next second, and the red point is the particle swarm distribution.

4. Resampling: The threshold of the number of particles is usually defined as Neff = N/2, where N is the initial number of particles. When the number of particles is less than Neff, re-sampling is required according to the weight.

5. Position estimation: Finally, the weighted average of all particles is used as the estimated position at the current moment.

The implementation details of fusion strategy are shown in Algorithm 1.
**Algorithm 1**: Integrated Positioning Strategy Based on Particle Filter**Input:** Particle range: Initial range x, Initial range y. The number of particles: N. Set particle moving direction: Random direction. Initialization weight: weights. Initialization stride: L.**Output:** Tracking results using particle filter and CNN model: state.  //$g\;\left(\delta \right)$ is the CNN positioning model, δ is real-time fingerprint grayscale image;1: **Initialization:** sample a set of particles from the initial state distribution2: **while** a new motion measurement **do**3: Current location update;// L is updated in real time according to the step stride estimation model (9).4:  **for** each particle **do**5:   Prediction: predict particle state by state transition Formula (8);6:   **if**
$g\;\left(\delta \right)=true$
**then //** CNN positioning results as observation results.**7:**     Update particle weights by Formula (10);8:   **else if** Particles through the wall (or building structure);9:    Update particle weights by map information (building boundaries, doors, windows, etc.);10:  **end for**11:  Weight: normalized;12:  **if** 1/sum(square(weights)) <length(particles)/2 **and** Carrier is in motion **then**13:    Resample: generate new particles based on their weights (multinomial resample);14:  **end if**15:  Update the Current location using states and weights.

## 5. Implementations and Evaluation

In this section, we mainly cover two parts of the experiment. First of all, in the first part, we verified the performance of the designed deep learning model, including model convergence speed and prediction accuracy, and compared it with commonly used machine learning models on the same dataset, and checked the effectiveness of the model. Then, test the positioning performance of the HPIPS system in different indoor scenarios. The specific test process is as follows.

**Figure 9:**
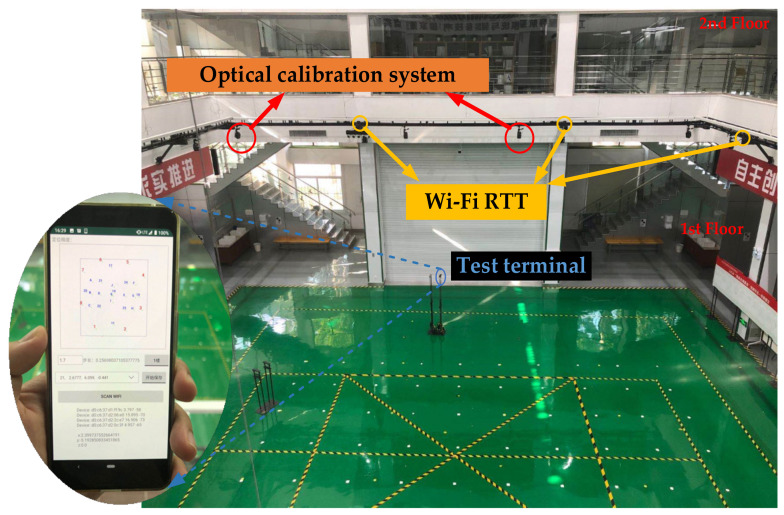
Positioning system test environment.

The test site shown in Figure 9, including the first and second floors, and the test area of each floor is about 16.9 m × 22.5 m. Eight APs are evenly deployed in the scene, and the base station deployment guidelines in Reference [42] are adopted. Since we only compare the horizontal positioning accuracy, the uniform distribution method is selected to have the smallest HDOP value and meet the principle of minimum error precision factor. In the test scenario, we planned the test path, including line-of-sight and non-line-of-sight parts, and selected some reference true values of total station calibration on the path to test the positioning accuracy. In the dynamic positioning test, the positioning terminal is tracked through a high-precision (mm-level) optical calibration system to complete real-time dynamic accuracy analysis. In general, the test environment we chose includes typical line-of-sight and non-line-of-sight parts and has test conditions to verify the performance of the proposed algorithm.

In the offline phase, the tester collects and records RTT ranging data every 1 m in the area to be located. The range value changes due to factors such as the signal receiving ability of each mobile terminal, the altitude, and the orientation of the AP. Therefore, when collecting data, five different types of mobile terminals are used, with a distance of 1.5, 1.7, and 2 m from the ground, and four orientations to collect data, to ensure the comprehensiveness of each sampling point. In the online phase, the tester holds the smartphone and walks along the planned route in the positioning area. At the same time, the optical calibration system tracks the position of the mobile phone. The positioning frequency of the mobile phone and the output frequency of the optical calibration system are the same. The positioning results are stored in the memory of the smartphone, to facilitate subsequent positioning performance analysis.

### 5.1. Analysis and Comparison of Model Performance

In order to verify the effectiveness of the proposed deep convolutional neural network model for fingerprint map positioning and the performance of the model on the constructed dataset, we set various hyperparameters as shown in Table 1.

The model is trained by setting the above hyperparameters and the constructed dataset. The performance of the model is analyzed from the four indicators of training accuracy, training loss, and test accuracy and test loss. The test results are as follows.

**Figure 10:**
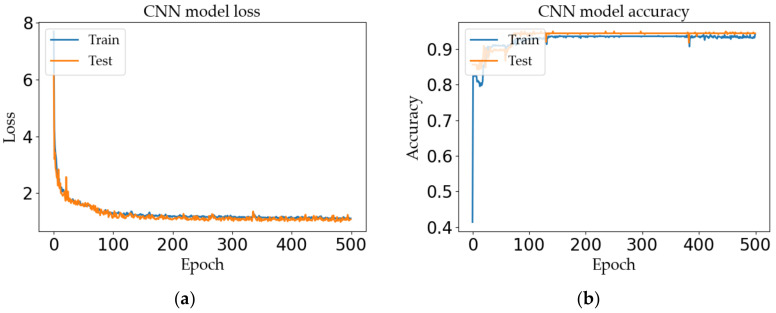
Performance analysis of positioning model: (**a**) loss curves and (**b**) accuracy curves.

It can be seen from Figure 10 that, after Epoch = 500 times, the initial loss of the model is reduced from 8.12 to 0.91, and the accuracy finally reaches 94.4%. It is verified that the model has high prediction accuracy for nonlinear and non-stationary ranging fingerprint signals, has good robustness, and can be applied to indoor location prediction.

**Figure 11:**
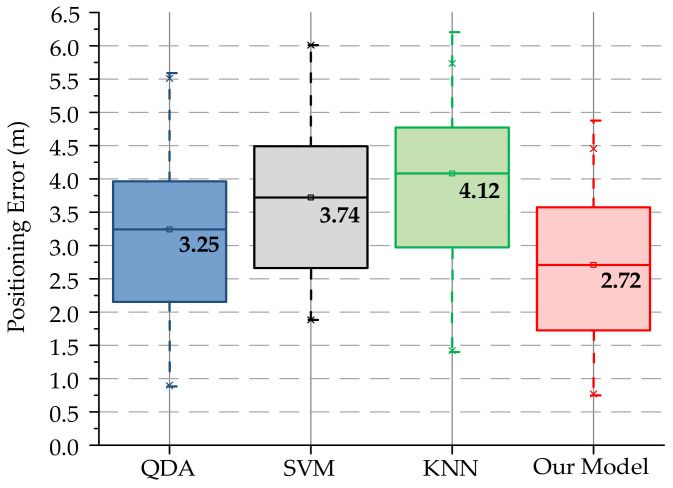
Comparative analysis of commonly used models. QDA, Quadratic Discriminant Analysis; SVM, Support Vector Machine; KNN, K Nearest Neighbors.

In order to verify the positioning performance of the proposed model, three commonly used machine learning classification models in the third-party machine learning module Scikit-learn are cited. Including Quadratic Discriminant Analysis (QDA), Support Vector Machine (SVM), and the most studied method in fingerprint positioning technology, K Nearest Neighbors (KNN). In QDA, the prior probability is set to “null”, the regularization parameter is 0, and the iterative convergence threshold is 0.0001. In SVM, the penalty coefficient C is set to 1, “Kernel” is set to the RBF function, the coefficient of “gamma” is set to the default value “auto”, and the degree is set to 3. In KNN, the positioning effect is obtained by comparing different K values, and finally K = 4 is determined by the Stratified k-fold cross validation score. In the test environment of Figure 9, the positioning accuracy test implemented, and the test result is shown in Figure 11.

The biggest advantage of box plots is that they are not affected by outliers and describe the discrete distribution of data in a relatively stable way, which can be used to analyze the positioning performance of different models. In Figure 11, the solid line in each box plot drawn represents the average value of the positioning error. Through comparison, it is found that the mean value of our proposed model is about 2.73 m, the first quartile is less than 2 m, and the span is relatively small. It performs better on the same dataset, which verifies the effectiveness of the model we designed and the method of constructing fingerprint grayscale images.

### 5.2. Multi-Source Fusion Location Experiment

In the above, we verified the effectiveness of the localization model based on the CNN network. The proposed CNN network model mainly provides a basis for the update of the observation equation in the particle filter algorithm. In this section, we will test the positioning performance of the positioning system fused with the CNN network model in the test scenario, and give clear test results through data analysis.

**Figure 12:**
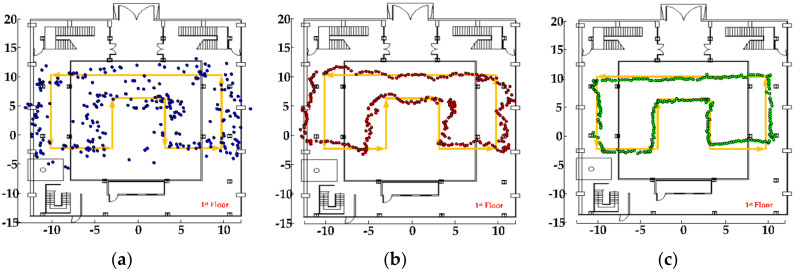
Positioning results in test environment 1: (**a**) CNN fingerprint positioning results, (**b**) particle filter (PF) fusion positioning results, and (**c**) PF fusion microelectromechanical systems (MEMS) + MAP positioning results.

(1) Test on the first floor of the test scene: The planned test route includes line-of-sight areas and non-line-of-sight areas. In non-line-of-sight, the signal has to pass through wooden boards and concrete walls to reach the receiving terminal. It can be known from Reference [43] that the signal loss of the template is about 1–1.8 dB, and the signal loss of the concrete wall is about 15–28 dB. Therefore, the signal fading in this environment is relatively large, and it is difficult to accurately and reliably locate through distance measurement. The tester holds a smartphone (the phone’s *z*-axis is perpendicular to the ground) and walks along the planned trajectory. At the same time, the optical calibration system is turned on to track the mobile phone. During the test, the pedestrian positioning results are recorded. The test track is shown in Figure 12.

In the figure above, the positioning trajectories of three positioning methods are drawn, and the yellow trajectory is the real planned path. The blue trajectory is the fingerprint positioning result of the CNN network model, and it can be seen that the positioning trajectory has a large fluctuation. Under the condition of line-of-sight and non-line-of-sight, the positioning result is relatively stable, which can basically reflect the walking trend of the tester. The red trajectory is the particle filter fusion CNN fingerprint positioning result, the trajectory is relatively smooth, and the fingerprint positioning result is used as an observation result to guide the movement of the particles, which can improve the positioning accuracy. The green trajectory is the particle filter fusion of the MEMS sensor information in the smart phone, which adapts to the particle movement step length, and uses the map information to dynamically update the weight information of the particles, which further improves the user’s freedom of movement and positioning accuracy.

**Figure 13:**
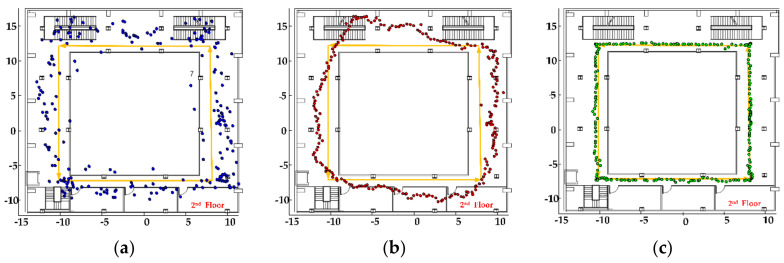
Positioning results in test environment 2: (**a**) CNN fingerprint positioning results, (**b**) PF fusion positioning results, and (**c**) PF fusion MEMS + MAP positioning results.

(2) Test on the second floor of the test scene: The characteristic of this test scene is that the signal passes through the glass to reach the receiving terminal. According to Reference [43], the loss of electromagnetic signals by glass is about 1.4–2.7 dB, which is usually isotropic and has relatively small influence. The experimental results are shown in Figure 13.

**Figure 14:**
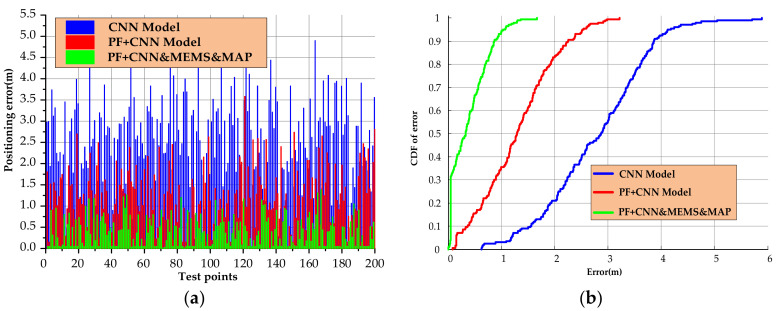
Error analysis of positioning results: (**a**) positioning error and (**b**) cumulative distribution function of error.

As shown in Figure 13, the yellow trajectory is the planned real trajectory. In Figure 13a, the middle blue trajectory is the fingerprint positioning result of the CNN model. It can be seen that there are large fluctuations and can follow the user to achieve rough positioning. In Figure 13b, the red trajectory is the result of particle filter fusion CNN fingerprint positioning. Its trajectory is relatively smooth, but part of the positioning result is outside the real scene. In Figure 13c, the green trajectory is the result of fusing the MEMS sensor and map information, constraining the movement state and weight of the particles, eliminating the particles passing through the wall, and achieving a better positioning effect. In order to compare the positioning performance of the positioning system more clearly, the errors of the positioning results in the two test environments were statistically analyzed, as shown in Figure 14.

It can be seen from Figure 14 that the blue curve is the positioning error using only the CNN model. The maximum positioning error is 4.9 m, the average positioning error is 2.58 m, and 59.4% of the errors are less than 3 m. After smoothing the positioning results of CNN with particle filter, the maximum positioning error is about 3.58 m, the average positioning error is 1.21 m, and 81.2% of the positioning errors are less than 2 m, which improves the positioning performance to a certain extent. The maximum positioning error of the HPIPS system proposed in this paper is only 1.38 m, and the average positioning error is 0.41 m, of which 94.2% of the errors are less than 1 m, achieving a better positioning effect. The specific positioning error statistics are shown in Table 2.

## 6. Discussion and Conclusions

A lightweight indoor positioning system based on smart phones was proposed, which can effectively solve the indoor positioning problems of pedestrians in line-of-sight and non-line-of-sight environments. First of all, in the first two sections of the article, the challenges faced by the current indoor positioning technology and common solutions were introduced. Then, in the third section, the composition framework and implementation process of the designed positioning system were introduced in detail. Then, in the fourth section, the implementation details of the algorithm were described in detail. First, the fingerprint location algorithm based on convolutional neural network was introduced, including the method of constructing dataset and the design of network model. Then, aiming at the problem of large fluctuations in positioning results, a positioning method based on adaptive particle filter fusion MEMS sensor and structured spatial information was proposed. It not only improves the degree of freedom when the positioning system is used, but also significantly improves the stability and positioning accuracy of the positioning results. In Section 5, the positioning performance of the proposed algorithm was verified through a large number of experiments. Within the coverage area, 94.2% of the positioning error is better than 1 m, and the average positioning error is 0.41 m. Therefore, as a lightweight, high-precision, and high-reliability positioning system, HPIPS has greater application and promotion value. In the research of this article, we did not choose the mainstream least square method based on distance measurement to solve the position, because factors such as signal multipath or occlusion often cause the algorithm to fail to converge.

In practical applications, the current positioning system still faces some challenges. For example, because the antenna of a smart phone is not isotropic, if we rotate the smart phone, the Wi-Fi RTT ranging information may change greatly, leading to increased positioning errors. Therefore, it is currently required that users who use the system must maintain a fixed posture, and cannot cover the antenna of the mobile phone during use. Because different mobile phones have different abilities to receive WIFI signals, the algorithm has poor adaptability to existing commercial mobile phones. In response to this problem, many scholars have carried out related research. For example, the authors of Reference [44] proposed a new method for establishing a fingerprint database which reduces the dependence of the device on the fingerprint database. We will continue to explore and solve the problem of differences in mobile-phone signal reception in subsequent research.

These problems seriously affect the user experience. Therefore, in the future work, it is an important aspect to solve the problem of the attitude of the smart phone in the user’s hands. Moreover, in the field of indoor positioning, the building structure in a structured environment is a kind of effective prior information, and how to make full use of this information is also our main future work. For example, as described in Reference [45], an RSSI map is established for each AP in combination with the map, and then the signal propagation model is calculated in the LOS and NLOS environment, thereby improving the positioning accuracy. The third point is to explore the use of deep learning and other methods to learn natural landmarks in the indoor environment, to correct the positioning results, and to achieve a lighter, more stable, and reliable low-cost indoor positioning solution which also has greater research significance.

## Figures and Tables

**Table 1:** Hyperparameters of the CNN model.

Hyperparameters	Values of Parameters
Input Size	25 × 25 (According to AP location)
Activation Function	ReLU (Rectified Liner Unit)
Number of Convolutional Layers	2
Pooling Size	2
Stride	1
Number of FC Layers	1
Optimizer	Adam
Learning Rate	0.001
Weight Decay	0.0005
Batch Size	50
Epochs	500

**Table 2:** Statistics of positioning errors.

Algorithm	CNN	PF + CNN	PF + CNN/MEMS/MAP
Mean Error (m)	2.58	1.21	0.41
65% Error (m)	3.25	1.54	0.52
Maximum Error (m)	4.90	3.58	1.38
